# Break-Induced Replication Is Highly Inaccurate

**DOI:** 10.1371/journal.pbio.1000594

**Published:** 2011-02-15

**Authors:** Angela Deem, Andrea Keszthelyi, Tiffany Blackgrove, Alexandra Vayl, Barbara Coffey, Ruchi Mathur, Andrei Chabes, Anna Malkova

**Affiliations:** 1 Department of Biology, School of Science, IUPUI, Indianapolis, Indiana, United States of America; 2 Department of Medical Biochemistry and Biophysics, Umeå University, Umeå, Sweden; 3 Laboratory for Molecular Infection Medicine Sweden (MIMS), Umeå University, Umeå, Sweden; National Cancer Institute, United States of America

## Abstract

DNA must be synthesized for purposes of genome duplication and DNA repair. While the former is a highly accurate process, short-patch synthesis associated with repair of DNA damage is often error-prone. Break-induced replication (BIR) is a unique cellular process that mimics normal DNA replication in its processivity, rate, and capacity to duplicate hundreds of kilobases, but is initiated at double-strand breaks (DSBs) rather than at replication origins. Here we employed a series of frameshift reporters to measure mutagenesis associated with BIR in *Saccharomyces cerevisiae*. We demonstrate that BIR DNA synthesis is intrinsically inaccurate over the entire path of the replication fork, as the rate of frameshift mutagenesis during BIR is up to 2,800-fold higher than during normal replication. Importantly, this high rate of mutagenesis was observed not only close to the DSB where BIR is less stable, but also far from the DSB where the BIR replication fork is fast and stabilized. We established that polymerase proofreading and mismatch repair correct BIR errors. Also, dNTP levels were elevated during BIR, and this contributed to BIR-related mutagenesis. We propose that a high level of DNA polymerase errors that is not fully compensated by error-correction mechanisms is largely responsible for mutagenesis during BIR, with Pol δ generating many of the mutagenic errors. We further postulate that activation of BIR in eukaryotic cells may significantly contribute to accumulation of mutations that fuel cancer and evolution.

## Abstract 2

DNA replication initiated by one-ended homologous recombination at a double-strand break is highly inaccurate, as it greatly stimulates frameshift mutations over the entire path of the replication fork.

## Author Summary

Accurate transmission of genetic information requires the precise replication of parental DNA. Mutations (which can be beneficial or deleterious) arise from errors that remain uncorrected. DNA replication occurs during S-phase of the cell cycle and is extremely accurate due to highly selective DNA polymerases coupled with effective error-correction mechanisms. In contrast, DNA synthesis associated with short-patch DNA repair is often error-prone. Break-induced replication (BIR) presents an interesting case of large-scale DNA duplication that occurs in the context of DNA repair. In this study we employed a yeast-based system to investigate the level of mutagenesis associated with BIR compared to mutagenesis during normal DNA replication. We report that frameshifts, which are the most deleterious kind of point mutation, are much more frequent during BIR than during normal DNA replication. Surprisingly, we observed that the majority of mutations associated with BIR were created by polymerases responsible for normal DNA replication, which are assumed to be highly precise. Overall, we propose that BIR is a novel source of mutagenesis that may contribute to disease genesis and evolution.

## Introduction

Genetic information is preserved through generations by chromosome duplication during S-phase DNA replication, which is highly accurate due to the fidelity of replicative polymerases and efficient elimination of replication errors by polymerase-coupled proofreading activity and post-replicative mismatch repair (MMR). Aside from scheduled DNA replication during S-phase, DNA synthesis is also a part of various types of DNA repair, such as nucleotide-excision repair, base-excision repair, and double-strand break (DSB) repair. It has been shown that short-patch synthesis associated with repair of various kinds of DNA damage is highly error-prone [1]–[5], making these events important contributors to a cell's overall mutation rate.

DSBs as a source of hypermutability have been documented for several repair events, including gene conversion (GC) and single-strand annealing in vegetative cells [4]–[9], and DSB repair in meiosis and non-dividing cells [10],[11]. Also, increased mutability has been associated with senescence in telomerase-deficient cells [12], where shortened chromosome ends behave similarly to DSB ends. At least two mechanisms were demonstrated to contribute to DSB-induced mutagenesis. First, unrepaired lesions accumulated in tracts of single-stranded DNA that form after a DSB result in error-prone restoration of the duplex molecule [9]. A similar pathway was shown to be responsible for hypermutagenesis associated with recovery of dysfunctional telomeres [9]. Second, it has been demonstrated that copying of a donor sequence associated with GC is mutagenic [5],[13],[14], which could be explained by inefficient MMR during GC [5],[6], or by an unusual, conservative mode of synthesis that proceeds without formation of a replication fork [15].

This study was designed to determine the mutation rate associated with a unique cellular process, break-induced replication (BIR), which is a processive type of DNA replication that can duplicate large chromosomal regions comparable in size to replicons. In stark contrast to S-phase replication, BIR is initiated at a DSB site rather than at a replication origin. BIR proceeds by invasion of one DSB end into the homologous template, followed by initiation of DNA synthesis that can continue for hundreds of kilobases. A variety of repair processes is believed to proceed via BIR, including repair of collapsed replication forks and stabilization of uncapped telomeres. BIR can also repair DSBs produced such that either only one of the two free DNA ends can find homology for strand invasion or both ends can find homology but only in different areas of the genome (reviewed in [16],[17]). Notably, a significant fraction of DSB gap repair events also proceed through BIR [18]. The occurrence of BIR often leads to loss of heterozygosity (LOH), chromosomal translocations, and alternative telomere lengthening [19]–[21], which are genetic instabilities associated with cancer in humans.

Unlike other forms of DSB repair, BIR is believed to proceed in the context of a replication fork [21], and the establishment of the BIR fork requires almost all of the proteins required for initiation of normal replication [22]. However, several observations indicate that the BIR replication fork may differ from an S-phase replication fork in several important ways. For example, it has been shown that, in *Saccharomyces cerevisiae*, BIR requires Pol32p, a subunit of polymerase δ (Pol δ; [21],[23],[24]) that is dispensable for yeast S-phase DNA replication. Further, the roles of the main replicative polymerases may differ between BIR and S-phase replication. Thus, for BIR initiation, only α-primase and Pol δ are essential, while polymerase ε (Pol ε) is involved only in later steps of BIR, and up to 25% of BIR events can complete in the absence of Pol ε [21]. Also, BIR initiation is very slow (takes approximately 4 h [18],[19],[23]) and is associated with frequent template switching that subsides after the first 10 kb of synthesis [25], which led to speculation that there may be slow assembly of an unstable replication fork that shifts to a more stable version later in synthesis. Alternatively, initiation of BIR might be slow due to a “recombination execution checkpoint” that regulates the initiation of DNA synthesis during BIR [18]. All of these unique features of BIR led us to test whether it is more mutagenic than S-phase replication.

Here we demonstrate that DNA synthesis associated with BIR is highly error-prone, as the frequency of frameshift mutations associated with BIR is dramatically increased compared to normal DNA replication. Our results indicate that BIR mutagenesis results from several problems, including increased polymerase error rate and reduced efficiency of MMR.

## Results

### Frameshift Mutagenesis Is Elevated during BIR

**Figure 1:**
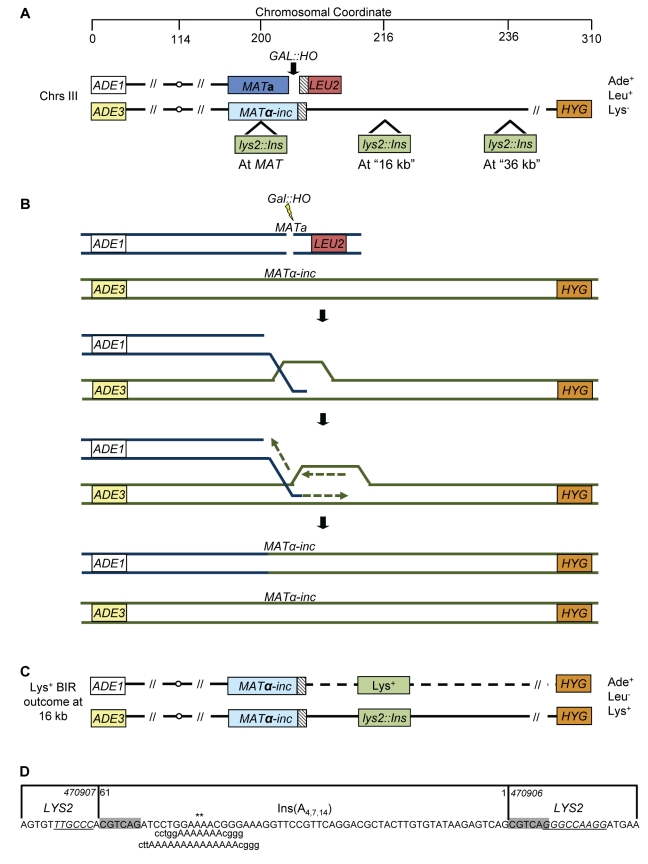
Experimental system to study BIR-associated mutagenesis. (A) Chromosome (Chr) III in a modified version of disomic experimental strain AM1003 [23] used to study BIR. A DSB is created at *MAT*a by a galactose-inducible *HO* gene. The *MAT*a-containing copy of Chr III is truncated by insertion of *LEU2* fused to telomere sequences, leaving only 46 bp of homology with the donor sequence (hatched rectangle). The *MATα*-*inc*-containing copy is full-length and is resistant to cutting by *HO*. In this strain, the majority of DSBs introduced at *MAT*a are repaired by BIR initiated by strand invasion centromere-proximal to *MATα-inc* followed by copying of the donor chromosome to the end. To assess mutagenesis associated with BIR, frameshift *lys2::Ins* reporters (see text for details) were inserted into donor Chr III at one of three positions located at different distances from *MATα-inc* (*MAT*, 16, or 36 kb). (B) After 5′-to-3′ resection, BIR proceeds through one-ended invasion of the broken molecule into the homologous donor chromosome. BIR-associated copying of approximately 100 kb of DNA from the donor chromosome results in an *α*-mating, Leu^−^ phenotype. (C) Frameshift mutations associated with BIR are detected by the Lys^+^ phenotype, which arises when an error in DNA copying that restores the *LYS2* reading frame is made in a second copy of the *lys2::Ins* reporter. The example depicts a Lys^+^ BIR event in the reporter at the 16 kb position. (D) Sequence of the 61, 64, and 70 bp inserts of the *lys2::Ins(A_4_)*, *lys2::Ins(A_7_)*, and *lys2::Ins(A_14_)* constructs, respectively, and flanking *LYS2* sequences. Asterisks indicate the location of the poly-A run; grey box shades the 6 bp direct repeats that flank the inserted sequence; nucleotides in underlined italics represent a mutation hotspot (right side) and its −1 bp quasipalindromic sequence (left side).

To assay the accuracy of BIR, we used a modified version of our disomic experimental system in *Saccharomyces cerevisiae* (Figure 1A), wherein a galactose-inducible DSB is initiated at the *MAT* **a** locus of the truncated, recipient copy of chromosome III, while the donor copy of chromosome III contains an uncleavable *MATα-inc* allele and serves as the template for DSB repair [23]. Elimination of all but 46 bp of homology on one side of the break on the recipient molecule via replacement with *LEU2* and telomeric sequences results in efficient DSB repair through BIR in this strain (Figure 1B,C). Initiation of BIR in this system is preceded by extensive 5′-to-3′ resection of the *GAL::HO-*induced DSB at *MAT* **a**, followed by strand invasion of the 3′ single-strand end into the donor chromosome at a position proximal to *MATα-inc* (Figure 1B; [26]). To study the accuracy of BIR, we chose to assay the level of frameshift mutagenesis using reversion frameshift reporters in our disomic strain. The frameshift reporters used allowed detection of mutations that occurred during BIR even in the presence of the original wild type gene (an essential feature because the wild type template allele remains after BIR repair) and also allowed us to investigate different aspects of BIR replication (similar to [27], see below). Frameshifts comprise a significant fraction (10%–20%) of all spontaneous mutations [28]–[30] and are the most deleterious type of point mutations, as they almost always eliminate gene function. In contrast, >90% of base substitutions are silent [31]. Notably, an increase in the rate of frameshifts typically correlates with an increase in base substitutions (reviewed in [32],[33]).

Three different frameshift reporters were employed: A_4_, A_7_, and A_14_ [27], which are all alleles of the *LYS2* gene with an insertion of approximately 60 bp that includes a homonucleotide run of four adenines (A_4_), seven adenines (A_7_), or 14 adenines (A_14_) (Figure 1D). Insertion of any of the three alleles results in a “+1” shift in the reading frame and a Lys^−^ phenotype, while a Lys^+^ phenotype is restored by a frameshift mutation that occurs in an approximately 71 bp region of the allele (that includes the inserted sequence) and restores the reading frame. A series of isogenic strains was created with insertion of the described reporter alleles into one of three positions on the donor (*MATα*-*inc*-containing) chromosome (Figure 1A): (1) at *MATα*-*inc* (“*MAT*”), (2) 16 kb centromere-distal from *MATα*-*inc* in the region between *RSC6* and *THR4* (“16 kb”), and (3) 36 kb centromere-distal to *MATα*-*inc* in the region between *SED4* and *ATG15* (“36 kb”). In all strains, *LYS2* was fully deleted from its native location in chromosome II (see Materials and Methods for details).

BIR-associated mutagenesis was measured by plating appropriate dilutions of cell suspensions to obtain single colonies on rich media (YEPD) and lysine drop-out media after a 7 h incubation in liquid galactose-containing media. The majority of cells undergoing DSB repair remained in G2/M arrest for the duration of the experiment (Figure S3A and unpublished data), consistent with repair of most DSBs by BIR, which exhibits delayed initiation associated with a long G2/M checkpoint arrest [19]. Coherently, the majority of colonies grown with or without selection (on lysine omission media or YEPD, respectively) repaired the DSB by BIR and displayed either an Ade^+^Leu^−^ or Ade^+/−^Leu^−^ phenotype (see Materials and Methods and Tables S1 and S2 for details), which were previously confirmed to result from BIR repair of both or one of two sister chromatids, respectively [23]. BIR efficiency in wild type and mutant strains is shown in Table S2.

**Figure 2:**
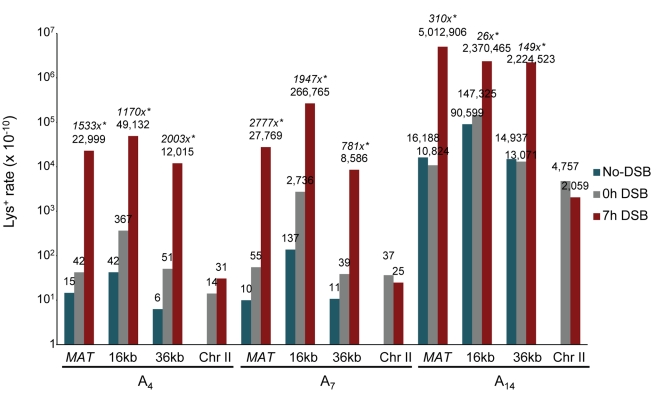
BIR-associated mutagenesis determined by frameshift reporters at three chromosomal positions. The rate of Lys^+^ revertants was measured before addition of galactose (0 h) and 7 h after incubation in galactose-containing media (7 h) in wild type and its various mutant derivatives containing frameshift reporters A_4_, A_7_, or A_14_ in the donor chromosome at *MATα-inc* (“*MAT*”) or approximately 16 or 36 kb centromere-distal to the DSB site. The rate of Lys^+^ revertants in strains with a DSB site in Chr III but containing frameshift reporters in the native *LYS2* position on chromosome II is also shown. Rates of spontaneous Lys^+^ mutagenesis were determined using isogenic no-DSB controls (“no-DSB”). Medians of mutation rates are plotted in log_10_ scale. See Table S1 for ranges of variation and numbers of repeats. Statistically significant differences from the rate of spontaneous events are indicated by *. The fold increase of the BIR mutation rate compared to spontaneous events is indicated in italics.

For all three reporters at all three locations, the rate of Lys^+^ frameshifts was much higher after DSB repair compared to the spontaneous Lys^+^ rate (Figure 2; Table S1; see Materials and Methods for details regarding rate calculations). Specifically, for all A_4_ and A_7_ strains, the rate of frameshift mutagenesis associated with DSB repair (7 h) exceeded the Lys^+^ reversion rate before the DSB (0 h) by 100- to 550-fold. Because most strains with a DSB site exhibited residual DSB formation even before addition of galactose (unpublished data), isogenic no-DSB controls were used to estimate more accurately the rate of spontaneous mutagenesis (see Materials and Methods for details). Using these no-DSB control strains lacking the *HO* cut site, we demonstrate a 780- to 2,800-fold increase in frameshift mutagenesis during BIR compared to spontaneous frameshift mutations. In all strains containing A_14_, in which spontaneous events were approximately 1,100- to 2,500-fold more frequent compared to A_4_, the rate of frameshift mutagenesis associated with DSB repair remained 25- to 300-fold higher than the rate of spontaneous events. Similar to unselected colonies, the majority of Lys^+^ DSB repair outcomes resulted from BIR (Table S1); thus, the substantial increase in frameshift mutagenesis observed in strains with DSBs compared to their no-DSB isogenic controls can be attributed to DNA synthesis during BIR. In control strains that contained the A_4_, A_7_, or A_14_ reporters in the native *LYS2* position on chromosome II, no increase in the rates of Lys^+^ was observed after 7 h in galactose (Figure 2; Table S1), which confirmed that the increased frameshift mutagenesis was specific for the chromosome undergoing BIR.

Lys^+^ BIR outcomes were primarily 1 bp deletions, the majority of which (70%–100%) occurred in ≥2 homonucleotide runs (Table 1; Figure S1). With data for all strains combined, the majority of Lys^+^ mutations concentrated in two hotspots: (1) the poly-A run, which is known to provoke replication slippage, and (2) the sequence GGGCCAAGG (Figures 1D and S1; Table 1), which could also promote replication slippage within one of its small homonucleotide runs. Alternatively, the second hot spot could result from template switching involving the first seven nucleotides of this hotspot (GGGCCAA) and its −1 bp quasipalindromic copy (TTGCCC) located approximately 70 bp away (Figures 1D and S1; see Discussion for details). As expected, the proportion of 1 bp deletions in the poly-A run increased with the length of the run, with only 3%, 20%, and 0% of frameshifts occurring in the poly-A run of the A_4_ reporter at the *MAT*, 16, and 36 kb positions, respectively, and 100% of frameshifts occurring in the A_14_ run at all three positions (Table 1 and Figure S1). The proportion of frameshifts in the A_7_ run varied somewhat across reporter positions. The spectra of Lys^+^ frameshift mutations were generally similar for BIR-induced compared to spontaneous mutations for each given reporter (Table 1 and Figure S1). One exception was the increase in 2 bp insertions observed in A_4_ and A_7_ no-DSB control strains at the 16 kb position. Taken together, our data show that frameshift mutagenesis during BIR is increased 25- to 2,800-fold compared to spontaneous mutagenesis.

**Table 1:** Spectrum of BIR-associated and spontaneous Lys^+^ mutations in MMR^+^ and *msh2Δ* strains.

Position	Reporter	*HO* Site	Genotype	1 bp Deletions			Other Mutations^a^	Total
				Poly-A Run	Other ≥2 nt Runs (HS)	Not in Run		
*MAT*	A_4_	DSB	WT	1	37 (35)	2	8	48
*MAT*	A_4_	No	WT	0	12 (12)	0	2	14
16 kb	A_4_	DSB	WT	10	33 (13)	6	10	59
16 kb	A_4_	No	WT	0	5 (2)	1	8	14
36 kb	A_4_	DSB	WT	0	35 (32)	15	2	52
36 kb	A_4_	No	WT	0	8 (8)	0	2	10
*MAT*	A_7_	DSB	WT	4	57 (57)	10	5	76
*MAT*	A_7_	No	WT	0	8 (8)	6	1	15
16 kb	A_7_	DSB	WT	47	3 (3)	1	1	52
16 kb	A_7_	No	WT	13	2 (1)	0	5	20
36 kb	A_7_	DSB	WT	4	26 (26)	3	3	36
36 kb	A_7_	No	WT	0	7 (7)	2	4	13
*MAT*	A_14_	DSB	WT	24	0 (0)	0	0	24
*MAT*	A_14_	No	WT	9	0 (0)	0	0	9
16 kb	A_14_	DSB	WT	24	0 (0)	0	1	25
16 kb	A_14_	No	WT	18	0 (0)	0	2	20
36 kb	A_14_	DSB	WT	14	0 (0)	0	0	14
36 kb	A_14_	No	WT	8	0 (0)	0	0	8
*MAT*	A_4_	DSB	*msh2Δ*	1	10 (10)	1	0	12
*MAT*	A_4_	No	*msh2Δ*	0	8 (8)	3	1	12
16 kb	A_4_	DSB	*msh2Δ*	10*	0 (0)	0	0	10
16 kb	A_4_	No	*msh2Δ*	12	0 (0)	1	0	13
36 kb	A_4_	DSB	*msh2Δ*	5*	12 (12)	0	0	17
36 kb	A_4_	No	*msh2Δ*	3	8 (8)	1	0	12
*MAT*	A_7_	DSB	*msh2Δ*	4*	6 (6)	1	0	11
*MAT*	A_7_	No	*msh2Δ*	18	0 (0)	0	1	19
16 kb	A_7_	DSB	*msh2Δ*	9	0 (0)	0	0	9
16 kb	A_7_	No	*msh2Δ*	17	0 (0)	0	0	17
36 kb	A_7_	DSB	*msh2Δ*	9*	7 (7)	0	0	16
36 kb	A_7_	No	*msh2Δ*	18	6 (6)	1	0	25

a Other mutations include insertions and >1 bp deletions, as well as 1 bp deletions accompanied by base substitutions;

*Percentage of 1 bp deletions occurring in the poly-A run is statistically significantly different from the isogenic wild type strain using Fisher's Exact Test (*p*<0.05). Abbreviations: bp, base pair; nt, nucleotide; HS, the GGGCCAAGG frameshift hotspot shown in Figure 1D and Table S1; DSB, double-strand break; WT, wild type.

### The Role of Translesion DNA Synthesis in BIR-Associated Mutagenesis

**Figure 3 pbio-1000594-g003:**
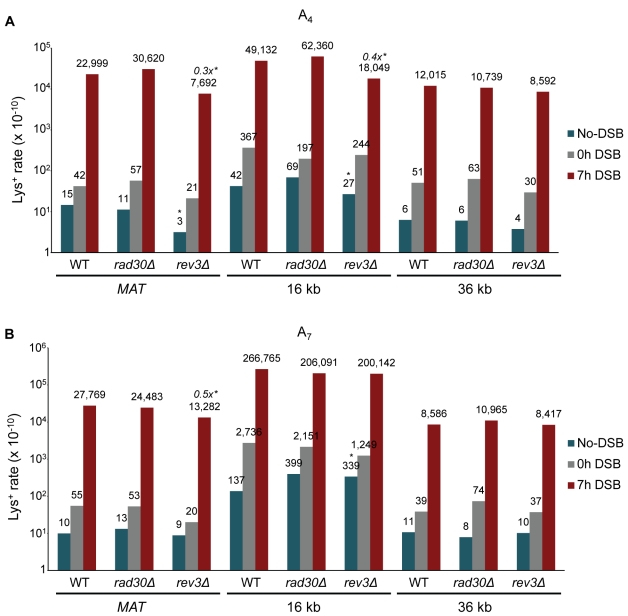
The role of translesion polymerases in BIR-associated mutagenesis. The rate of Lys^+^ revertants was measured before addition of galactose (0 h) and 7 h after incubation in galactose-containing media (7 h) in wild type and its *rad30Δ* (Pol η-deficient) and *rev3Δ* (Pol ζ-deficient) mutant derivatives containing frameshift reporters (A) A_4_ or (B) A_7_ at the *MAT*, 16 kb, and 36 kb chromosomal positions. Statistically significant differences from the rate of wild type events are indicated by *. The fold increase of BIR mutation rate in mutants compared to wild type (in cases of a statistically significant change) is indicated in italics. Other abbreviations and statistical details are similar to those provided in the legend of Figure 2.

We hypothesized that involvement of translesion DNA synthesis during BIR, whether due to a defective replisome or DNA template damage (as discussed in [4],[9],[34],[35]), may contribute to the increased rate of BIR frameshift mutations. To address this, BIR-associated mutagenesis in A_4_ and A_7_ strains with deletion of *RAD30* (encoding DNA polymerase η [Pol η]) or deletion of *REV3* (encoding the catalytic subunit of DNA polymerase ζ [Pol ζ]) was measured at all three positions (Figure 3A,B; Table S1). While *rad30Δ* mutants showed no change in the rate of frameshift mutations compared to wild type at any position, deletion of *REV3* did result in a small but statistically significant decrease (2×–3×) in mutations at *MAT* and for A_4_ at 16 kb. No change was observed in the other *rev3Δ *strains. (Importantly, BIR efficiency in *rev3Δ* mutants was similar to that observed in wild type [Table S2 and unpublished data]).

To differentiate the role of *REV3* in BIR from its role in damage-induced mutagenesis, we exposed our *rev3Δ* no-DSB control strains containing A_7_ at the 36 kb position (where there was no effect of *rev3Δ* on BIR mutagenesis) to 20 J/m^2^ of UV light (Figure S2). This exposure resulted in an approximately 10-fold increase in Lys^+^ events compared to the frequency of spontaneous events. Consistent with the observation of Abdulovic and Jinks-Robertson [36], the UV-induced increase in mutagenesis was largely *REV3*-dependent in our system. Thus, we conclude that BIR-induced mutagenesis differs from UV-induced mutagenesis in its dependency on Pol ζ; while the latter strongly depends on Pol ζ, the former is only modestly dependent on Pol ζ and only at some chromosomal positions.

### The Role of MMR in BIR-Associated Hypermutability

**Figure 4:**
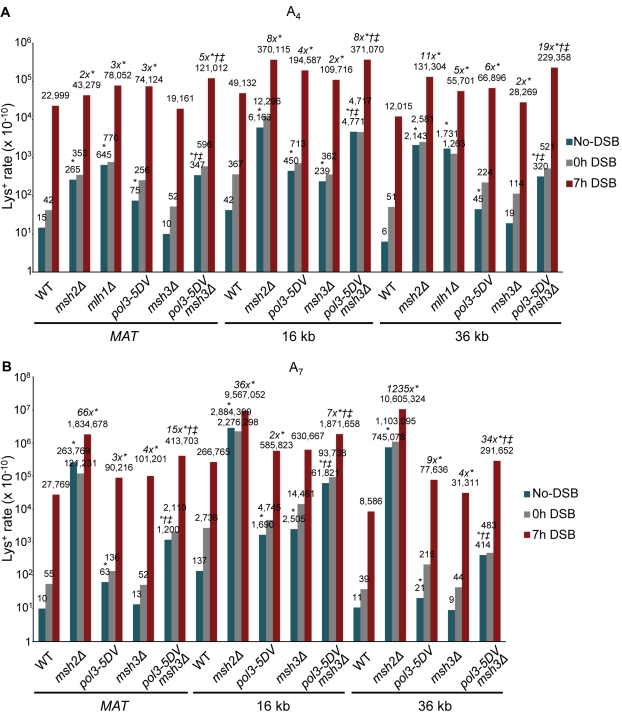
The role of MMR and polymerase proofreading in BIR-associated mutagenesis. The rate of Lys^+^ revertants was measured before addition of galactose (0 h) and 7 h after incubation in galactose-containing media (7 h) in wild type and its various MMR^−^ and Pol δ proofreading-deficient derivatives containing frameshift reporters (A) A_4_ or (B) A_7_ at the *MAT*, 16 kb, and 36 kb chromosomal positions. Statistically significant differences from wild type are indicated by *. The fold increase of BIR mutation rate in mutants compared to wild type (in cases of a statistically significant change) is indicated in italics. Statistically significant differences from *msh3Δ* are indicated by ^†^. Statistically significant differences from *pol3-5DV* are indicated by ^‡^. Other abbreviations and statistical details are similar to those provided in the legend of Figure 2.

We eliminated *MSH2* from all A_4_ and A_7_ strains to test whether MMR corrects frameshift errors made during BIR. In all cases, we observed a significant increase in frameshift mutagenesis during BIR in *msh2Δ* strains compared to their isogenic wild type strains, suggesting that MMR corrects a large number of BIR frameshift errors (Figure 4A,B; Table S1). The mutation rate observed during BIR in MMR-deficient mutants significantly exceeded the level of spontaneous mutagenesis observed in MMR-deficient no-DSB controls, confirming that MMR deficiency further increased the already high rate of BIR mutagenesis. Strains containing A_7_ reporters were more sensitive to MMR deficiency and showed higher increases in the rate of frameshifts compared to increases for the corresponding A_4_ strains. This effect is similar to the effect of *msh2Δ* during normal replication, where MMR is especially important to correct errors in long homonucleotide runs [27],[37],[38]. Also, consistent with Tran et al. [27], who reported a dramatic shift of spontaneous frameshifts to the poly-A run in MMR-deficient A_7_ strains, we observed a significantly higher percentage of mutations occurring in the poly-A run in MMR-deficient A_7_ strains at the *MAT* and 36 kb positions compared to isogenic MMR-proficient strains (Table 1; Figure S1). At the 16 kb position, where most events in the wild type A_7_ strain were in the poly-A run, we confirmed that MMR deficiency caused mutation events to shift to the poly-A run in the A_4_ strain. The ability of MMR to correct BIR errors was further supported by data from *mlh1Δ* mutants, which were tested at the *MAT* and 36 kb positions with the A_4_ reporter (Figure 4A; Table S1). Our data thus suggest that BIR occurs in the context of functional MMR machinery and that long homonucleotide runs are especially susceptible to failure of MMR, as is the case during normal DNA replication.

To better characterize the role of MMR in correction of BIR errors, we compared mutation rates in experimental MMR-deficient strains with their no-DSB controls. This comparison showed that, prior to MMR correction, the level of polymerase errors was significantly higher during BIR compared to normal DNA replication for all constructs (Table 2). Based on the percent of these errors that was repaired by MMR (calculated in Table 2), the efficiency of MMR in BIR was 98%, 97%, and 99.9% for A_7_ strains at the *MAT*, 16, and 36 kb positions, respectively, and approached 99.9% for all positions during normal DNA replication. MMR also repaired a high percentage of BIR errors in A_4_ strains (47%, 87%, and 91% at the *MAT*, 16, and 36 kb positions, respectively), but this was somewhat lower than the efficiency of MMR during normal DNA replication for these strains (94%, 99%, and 99.7% at the MAT, 16, and 36 kb positions, respectively). These data suggest that, although MMR operates during BIR, the percentage of MMR-repaired polymerase errors is often lower for BIR than for normal replication.

**Table 2:** Efficiency of MMR during BIR repair.

Position	Reporter	Type of DNA Synthesis	Polymerase Errors				Fold Increase in Errors Before vs. After MMR Repair^e^
			Total Before MMR Repair (per 10^10^)^a^	Total After MMR Repair (per 10^10^)^b^	Total Repaired by MMR (per 10^10^)^c^	% Repaired by MMR^d^	
*MAT*	A_4_	S-phase	265	15	250	94.34	17.6
*MAT*	A_4_	BIR (BIR/S-phase)	43,279 (163)	22,999 (1,533)	20,280	46.86	1.8
16 kb	A_4_	S-phase	6,163	42	6,121	99.32	147.0
16 kb	A_4_	BIR (BIR/S-phase)	370,115 (60)	49,132 (1,170)	320,983	86.73	7.5
36 kb	A_4_	S-phase	2,143	6	2,137	99.72	357.2
36 kb	A_4_	BIR (BIR/S-phase)	131,304 (61)	12,015 (2,003)	119,289	90.85	10.9
*MAT*	A_7_	S-phase	263,769	10	263,759	99.996	26,376.9
*MAT*	A_7_	BIR (BIR/S-phase)	1,834,678 (7)	27,769 (2,777)	1,806,909	98.486	66.1
16 kb	A_7_	S-phase	2,884,399	137	2,884,262	99.995	21,054.0
16 kb	A_7_	BIR (BIR/S-phase)	9,567,052 (3)	266,765 (1,947)	9,300,287	97.211	35.9
36 kb	A_7_	S-phase	745,078	11	745,067	99.999	67,734.4
36 kb	A_7_	BIR (BIR/S-phase)	10,605,324 (14)	8,586 (781)	10,596,738	99.919	1,235.2

a Rate of Lys^+^ frameshifts in *msh2Δ*;

b Rate of Lys^+^ frameshifts in wt;

c (Total before MMR repair) − (Total after MMR repair);

d (Total repaired by MMR)/(Total before MMR repair);

e (Total before MMR repair)/(Total after MMR repair). Abbreviations: BIR, break-induced replication; MMR, mismatch repair.

### The Role of Polymerase Proofreading in BIR-Associated Mutagenesis

Our previous analysis indicated that BIR is associated with a significantly higher level of polymerase errors than normal replication (see above). To determine the role of proofreading activity during BIR, *pol3-5DV*, an exonuclease-deficient mutation, was introduced into A_4_ and A_7_ strains at all three chromosomal locations to eliminate the proofreading activity of Pol δ (Figure 4A,B; Table S1). Pol δ was chosen because, unlike Pol ε, it is required at all stages of BIR synthesis [21]. *pol3-5DV* strains consistently showed an increase in BIR-related mutagenesis above the already high mutagenesis observed in wild type strains (a 3- to 6-fold increase for A_4_ and a 2- to 9-fold increase for A_7_). Also, the frameshift mutation spectrum of Lys^+^ outcomes in *pol3-5DV* strains was similar to that in their respective wild type strains (unpublished data). These results indicate that the proofreading activity of Pol δ is capable of correcting polymerase errors made during BIR.

The role of proofreading activity can be accurately estimated only in the absence of MMR due to redundancy between the two activities. However, haploid *pol3-5DV* mutants are inviable in combination with full MMR deficiency; thus, we deleted *MSH3* (which is known to result in a partial MMR defect) in *pol3-5DV* strains to better understand the effect of Pol δ proofreading activity. (The growth rate of *pol3-5DVmsh3Δ* double mutants was similar to wild type [Figure S3B,C] and its viability was not reduced following 7 h incubation in galactose [unpublished data]). We observed synergistic increases in BIR frameshift mutagenesis in *pol3-5DVmsh3Δ* double mutants compared to their respective single-mutant strains at all positions (Figure 4A,B; Table S1). However, the increase in mutagenesis that resulted from synergism between *pol3-5DV* and *msh3Δ* was generally higher for spontaneous events compared to BIR events, which may indicate decreased efficiency of Pol δ proofreading during BIR. Finally, because the increase in mutagenesis was observed in a mutant lacking the exonuclease activity of Pol δ many replication errors during BIR must be produced by Pol δ [39], although it cannot be excluded that DNA synthesis errors by other polymerases contribute as well [40],[41].

### Elevated dNTP Pools Contribute to Impaired Polymerase Fidelity Associated with BIR

**Figure 5:**
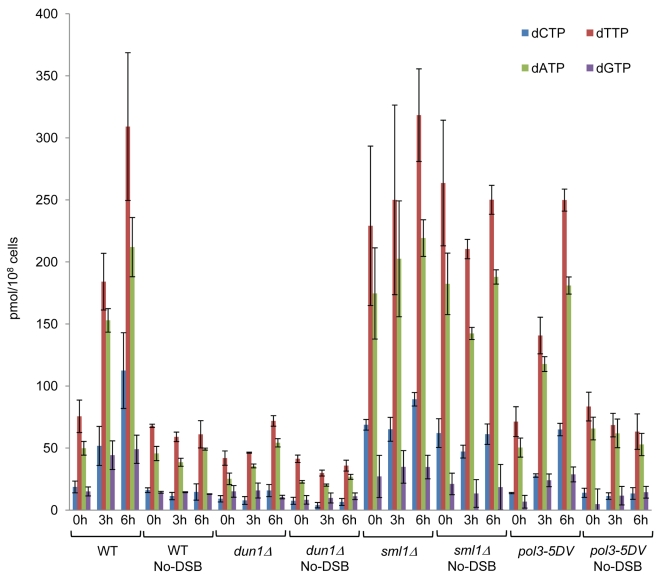
BIR is associated with increased dNTP levels. dNTP levels measured in strains containing the A_4_ reporter at the 16 kb position before addition of galactose (0 h) and after 3 h and 6 h of incubation in galactose-containing media. dNTP levels were measured in wild type and its *dun1Δ*, *sml1Δ*, and *pol3-5DV* derivatives containing a DSB site, as well as in their respective no-DSB control strains. dNTP levels are presented as the average ± standard deviation of three time course experiments for each strain.

Previously, we established that BIR proceeds under conditions of G2/M cell-cycle arrest resulting from the DNA damage checkpoint response [19],[23], and we hypothesized that cells with chromosome(s) undergoing BIR repair may induce ribonucleotide reductase (RNR) and dNTP levels in a manner similar to other damage-induced checkpoint responses ([42], and reviewed in [43]). To test this hypothesis, we employed the strain with the A_4_ reporter at the 16 kb position to measure dNTP pools before galactose induction of the DSB (0 h), and 3 and 6 h after galactose addition (Figure 5). (BIR-associated DNA synthesis is initiated approximately 4–6 h after galactose addition [19],[23].) Compared to pre-induction levels, a 2- to 3-fold increase in dNTP levels was evident at the 3 h time point, and a 3- to 6-fold increase was observed at the 6 h time point (Figure 5). No increase in dNTP levels was observed at either the 3 or 6 h time points in the isogenic no-DSB control strain. Also, we observed increased levels of the DNA-damage inducible RNR subunits Rnr2p and Rnr4p and decreased levels of RNR inhibitor Sml1p in cells undergoing BIR (Figure S4). In the absence of Dun1p, which is required for degradation of Sml1p and induction of RNR genes in other cases of DNA-damage response [44],[45], the increase in dNTP levels was nearly eliminated during BIR repair. A 1.6- to 2.1-fold reduction in dNTP levels was observed in the *dun1Δ* no-DSB control strain compared to the wild type no-DSB strain (Figure 5). Finally, deletion of *SML1*, which is known to increase basal dNTP levels [46], did not affect dNTP levels during BIR, but the *sml1Δ* mutation in no-DSB control strains did produce an expected increase in dNTP levels. These data are consistent with the roles of Dun1p and Sml1p in the regulation of dNTP levels in vegetative cells and demonstrate that BIR occurs in the context of *DUN1*-dependent up-regulation of RNR activity.

**Figure 6:**
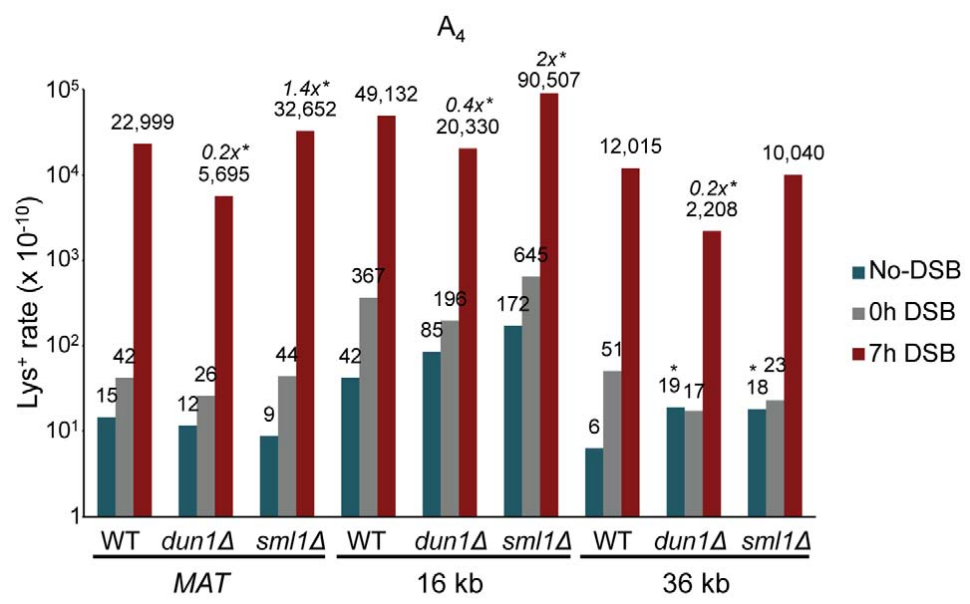
Effect of mutations affecting dNTP levels on BIR-associated mutagenesis. The rate of Lys^+^ revertants was measured before addition of galactose (0 h) and 7 h after incubation in galactose-containing media (7 h) in wild type and its *dun1Δ* and *sml1Δ* derivatives containing the A_4_ frameshift reporter at the *MAT*, 16 kb, and 36 kb chromosomal positions. Statistically significant differences from the rate of wild type events are indicated by *. The fold increase of BIR mutation rate in mutants compared to wild type (in cases of a statistically significant change) is indicated in italics. Other abbreviations and statistical details are similar to those provided in the legend of Figure 2.

Increased dNTP levels are known to decrease the fidelity of DNA polymerases and are associated with increased mutation rates. To investigate the role of increased nucleotide pools in BIR-related mutagenesis, the level of frameshift mutagenesis was measured in *dun1Δ* and *sml1Δ* A_4_ strains (Figure 6; Table S1). BIR mutagenesis decreased by 4.0-, 2.4-, and 5.4-fold at the *MAT*, 16, and 36 kb positions, respectively, in *dun1Δ* compared to wild type. The efficiency of BIR in *dun1Δ* cells was slightly reduced (a 1.2-fold reduction at all positions; Table S2 and unpublished data) compared to wild type. This decrease in BIR efficiency results most likely from a checkpoint response deficit in *dun1Δ*, which may lead to premature recovery from G2 arrest of cells undergoing BIR repair and, therefore, to increased loss of the broken chromosome due to mis-segregation. To accommodate this, the data were re-calculated to determine the rate of Lys^+^ events per BIR event. These results confirm that the *dun1Δ* mutation reduced the rate of frameshift mutations by 3.3-, 2.0-, and 4.8-fold at the *MAT*, 16, and 36 kb positions, respectively. Conversely, *sml1Δ* mutants did not display any change in the rate of mutations associated with BIR at the 36 kb position but did show small, 1.4-, and 1.8-fold increases at *MAT* and 16 kb.

We propose that increased dNTP pools contribute to the high mutagenesis associated with BIR. However, the fact that mutagenesis during BIR remained approximately 100- to 500-fold higher than during normal DNA synthesis even in *dun1Δ* mutants suggests that a large portion of BIR-related mutations may be independent of dNTP levels (see Discussion). Importantly, we tested the effect of the *pol3-5DV* mutation on dNTP levels both during BIR and in no-DSB controls and confirmed that this mutation did not affect dNTP levels in either case (Figure 5), consistent with our prior conclusion that the observed effects of *pol3-5DV* on BIR-induced mutagenesis resulted directly from the proofreading defect.

## Discussion

### BIR Is a Versatile Frameshift Mutagen

The fidelity of DNA synthesis differs among the various processes in which it is involved. While replication accomplishing genomic duplication is highly accurate, short-patch synthesis associated with repair of DNA damage, such as repair of DSBs by GC, is error-prone. In this study, we demonstrate that BIR, which can duplicate replicon-sized regions of chromosomes and is believed to proceed in the context of a replication fork [21], is associated with frameshift mutation rate increases up to 2,800-fold compared to spontaneous events. BIR-related hypermutability persisted at sites 16 and 36 kb distal to the DSB, differentiating this mechanism from the “template-switching” phenomenon discovered by Smith et al. [25] that was detected only within the first 10 kb of the 97 kb template that was copied.

Overall, the frameshift spectrum observed in *lys2::Ins* reporters during BIR was similar to the spectrum of spontaneous frameshifts for the same reporters/positions, with the majority of events occurring in di-, tri-, or poly-nucleotide runs. Also, BIR did not cause large deletions such as those increased in the *pol3-t* mutant, where they were explained by template switching between direct repeats stimulated by formation of extensive regions of ssDNA [47]. For both BIR-related and spontaneous events, two locations were especially susceptible to frameshift mutations. First, as previously described [27], the poly-A run induced frameshift mutations in a length-dependent manner. A second hotspot, the sequence GGGCCAAGG, may promote frameshift errors due to its multiple polynucleotide repeats, or as a result of template switching between this sequence and its partial −1 bp quasipalindromic copy (TTGCCC) located approximately 70 bp away. The mechanism of brief template switching of the nascent DNA strand to a nearly identical, inverted sequence was first proposed by Lynn Ripley [48] and later reported by Strathern et al. [4] and Hicks et al. [5], who observed alteration of nearly palindromic sequences into perfect palindromes during DSB repair by GC. However, in their systems, the inverted repeats were in much closer proximity than in our strains. The overall similarity between spontaneous and BIR-related frameshift mutation spectra observed in our assay could reflect that the nature of mutations is similar for both BIR- and S-phase replication. A second interesting possibility is that inter-sister BIR contributes to spontaneous events.

### Increased dNTP Pools Are a Prominent Source of BIR-Associated Mutagenesis

Cells undergoing BIR repair arrest as a part of the G2/M DNA damage checkpoint response [19]. Here, we observed that this checkpoint response also leads to an increase of Rnr2p and Rnr4p, a decrease of Sml1p, and increased dNTP pools, which require the checkpoint kinase Dun1p. Dun1p is a downstream target of the Mec1p and Rad53p checkpoint pathway that activates RNR by multiple mechanisms (reviewed in [49]). Because induction of RNR is considered the last step in the checkpoint cascade, our data suggest that a single DSB undergoing BIR repair triggers a complete checkpoint response (which includes both cell cycle arrest and RNR induction), which differentiates this process from the truncated checkpoint response observed, for example, in yeast undergoing DSB repair in G1 [50].

The significant decrease in BIR mutagenesis observed in *dun1Δ* suggests that increased dNTPs contribute to BIR-induced mutagenesis. Nevertheless, even in *dun1Δ*, BIR mutagenesis remained at least 100-fold higher than the spontaneous level of mutations, which indicates that elevated dNTP pools alone cannot explain the decrease in replication fidelity. These findings are consistent with previous work that has shown elevated dNTP levels to be mildly mutagenic in the presence of MMR [42],[51], and we propose that the role of elevated dNTP pools in our system is to further increase the number of errors made by an already error-prone fork, as discussed below. As expected, *sml1Δ* increased basal levels of dNTPs at 0 h but did not affect post-DSB increases because, in wild-type cells, Sml1p is degraded during BIR (Figure S4). Consistently, *sml1Δ* did not change the level of BIR mutagenesis at 36 kb; however, *sml1Δ* resulted in small but significant increases in BIR mutagenesis at the *MAT* and 16 kb positions. The reason for these increases is unclear, but exposure of the cell to chronically elevated levels of dNTPs may play a role.

### BIR-Associated Mutagenesis Is Due to Uncorrected Errors of the Replication Fork

Mutations arise from two sources: uncorrected replication errors left by a replication fork copying an undamaged template and error-prone copying of damaged DNA by a translesion polymerase. We investigated the role of translesion-synthesis polymerases Pol η and Pol ζ in BIR mutagenesis and determined that hypermutability during BIR is independent of Pol η, while modestly dependent on Pol ζ at some chromosomal positions. The activity of Pol ζ is known to be highly mutagenic in yeast, with the majority of damage-induced and over half of spontaneous mutations ascribed to Pol ζ, whereas lesion bypass by Pol η can be error-free or error-prone depending on the type of lesion and experimental assay employed (reviewed in [34],[52]). Here we observed no effect of *rad30Δ* on BIR-related mutagenesis. At the 36 kb position, where BIR is fast and stable, BIR-mutagenesis was also *REV3*-independent. In contrast, UV-induced mutagenesis at the 36 kb position was largely *REV3*-dependent (consistent with data in [36]), emphasizing the difference in the role of Pol ζ in BIR versus damage-induced mutagenesis. Interestingly, a small but significant reduction in BIR mutations occurred in *rev3Δ* mutants at *MAT*. One possible explanation is that the slow initiation of BIR in this region [18],[19] results in persistent ssDNA in the D-loop, which leads to higher mutagenesis, presumably by accumulating endogenous damage in ssDNA. Previously, increased spontaneous mutagenesis in regions of artificially created transient ssDNA at DSBs and uncapped telomeres was shown to significantly decrease in the absence of *REV3* [9]. Pol ζ dependence was also observed at the 16 kb position in the A_4_ strain. This location may be more difficult for replication machinery to traverse, as evidenced by the overall increased rate of mutations (both spontaneous and BIR-related) at this position compared to others. Lack of Pol ζ dependence for the A_7_ construct at the 16 kb position could be explained by additional mutations in the poly-A run, which could be Pol ζ-independent. Overall, we conclude that BIR-associated frameshift mutagenesis is independent of Pol η, while modestly dependent on Pol ζ at some chromosomal positions.

We found that MMR operates during BIR but is often less efficient at correcting BIR-related versus spontaneous errors. This could indicate a decreased efficiency of MMR to correct any individual error made during BIR or that the amount of errors during BIR is sufficiently high to overwhelm MMR repair capabilities. Alternatively, it could indicate that BIR mutants result from both MMR-dependent and MMR-independent pathways, as has been proposed for spontaneous mutations [47]. This final possibility is supported by our observation of higher effects of *msh2Δ* in A_7_ versus A_4_ strains, because the increased number of errors in A_7_ results from replication slippage in the poly-A run, which is efficiently repaired by MMR [27]. The varying ratio of MMR-dependent to MMR-independent mutation events may explain the varying effect of *msh2Δ* across the three chromosomal locations on both BIR-associated and spontaneous mutagenesis (Table 2), as well as the context-dependence of MMR previously observed by Hawk et al. [53] for spontaneous mutagenesis. In *pol3-5DV* mutants, in which Pol δ proofreading was inactivated, we observed a further increase in the mutation rate compared to wild type, suggesting that proofreading activity operates during BIR. This result implicates Pol δ as one polymerase responsible for many BIR elongation errors [39], although other polymerases may contribute as well [40],[41]. The synergistic increase in BIR mutations observed in *pol3-5DVmsh3Δ* double mutants further supports the involvement of Pol δ proofreading during BIR. However, the efficiency of Pol δ proofreading of BIR errors appeared somewhat lower compared to S-phase replication. Furthermore, this synergism suggests that Pol δ introduces mutagenic errors during BIR replication associated with MMR, versus during other repair-related synthesis.

In summary, we propose that the high level of BIR-associated frameshift mutagenesis is due to uncorrected errors left by a mutagenic replication fork. Our data suggest that undamaged template DNA is copied by a BIR fork that contains multiple deficiencies, including decreased Pol δ replication fidelity in the presence of increased nucleotide pools and reduced MMR efficiency, which act synergistically to markedly increase frameshift mutagenesis. This proposed mechanism is generally similar to the mechanism recently suggested to generate mutations during GC repair [5]. What is unexpected is to observe such similar mutation mechanisms between GC, which proceeds through synthesis-dependent strand annealing that does not assemble a replication fork [15], and BIR, which proceeds in the context of a replication fork [21],[22].

### Genome Destabilization by BIR-Associated Mutagenesis

BIR has been documented in both prokaryotes and eukaryotes and has been implicated in various processes of DNA metabolism. BIR is believed to restart collapsed replication forks, which occur even in healthy, dividing cells (reviewed in [17]), and it is also required for telomere maintenance in telomerase-deficient cells [21]. Also, a significant fraction of DSB gap repair events has been shown to proceed through BIR [18]. BIR leads to non-reciprocal translocations similar to those leading to cancer and other human diseases [54],[55]. It has been demonstrated that translocations mediated by BIR are often initiated by DSBs introduced near transposons or other DNA repeats that are present at multiple genomic locations [56]. A BIR-like repair pathway, microhomology-mediated BIR, was reported to generate copy number variations in eukaryotes, including those leading to human disease [57],[58]. Based on its widespread involvement in various processes, we propose that BIR may significantly contribute to the mutation rate and spectrum of many cell types, which is relevant to both disease development and selective adaptation. It may also provide an additional mechanism for so-called “mutation showers” reported to contribute to up to 1% of all mutations in the mouse genome [59]. BIR-associated mutagenesis may have an especially important role in tumorigenesis, because human cancer cells may both activate BIR at an elevated rate and be MMR-deficient (reviewed in [60]). Also, several human tumor-suppressor genes contain homonucleotide runs [61]–[64], which we demonstrated confer hypermutability in the context of BIR in MMR-deficient cells.

## Materials and Methods

### Yeast Strains

All yeast strains were isogenic to AM1003 [23], which is a chromosome III disome with the following genotype: *hmlΔ::ADE1/hmlΔ::ADE3 MAT* **a**-*LEU2*-tel/*MATα-inc hmrΔ::HYG* FS2*Δ*::*NAT*/FS2 *leu2/leu2-3,112 thr4 ura3-52 ade3::GAL::HO ade1 met13*. In this strain, the *HO* endonuclease-induced DSBs introduced at *MAT* **a** are predominantly repaired by BIR because the portion of the chromosome centromere-distal to *MAT* **a** is truncated to leave only 46 bp of homology with the donor sequence [19],[23]. All strains used for measuring mutagenesis were constructed using PCR-based gene disruption and direct genome modification by oligonucleotides as described (see Text S1 for details) [65],[66]. All single-gene deletion mutants were constructed by transformation with a PCR-derived *KAN-MX* module flanked by terminal sequences homologous to the sequences flanking the open reading frame of each gene [67]. All constructs were confirmed by PCR and by phenotype. Proofreading-deficient mutant *pol3-5DV* was constructed as described [39] and confirmed by PCR followed by restriction analysis with *Hae*III. Control no-DSB strains were obtained from each experimental strain by plating on YEP-Gal media, followed by selection of Ade^+^Leu^+^ colonies resulting from GC repair of the DSB at *MAT* **a**.

### Media and Growth Conditions

Rich medium (yeast extract-peptone-dextrose [YEPD]) and synthetic complete medium, with bases and amino acids omitted as specified, were made as described [68]. YEP-lactate (YEP-Lac) and YEP-galactose (YEP-Gal) contained 1% yeast extract and 2% Bacto peptone media supplemented with 3.7% lactic acid (pH 5.5) or 2% (w/v) galactose, respectively. Cultures were grown at 30°C.

### Mutagenesis Associated with DSB Repair

To determine mutation frequency, yeast strains were grown from individual colonies with agitation in liquid synthetic media lacking leucine for approximately 20 h, diluted 20-fold with fresh YEP-Lac, and grown to logarithmic phase for approximately 16 h. Next, 20% galactose was added to the culture to a final concentration of 2%, and cells were incubated with agitation for 7 h. No-DSB control strains were subjected to the same incubation and plating processes.

Samples from each culture were plated at appropriate concentrations on YEPD and lysine drop-out media before (0 h) and 7 h after the addition of galactose (7 h) to measure the frequency of Lys^+^ cells. Because spontaneous mutation frequencies were calculated based on the number of mutations accumulated during many cell generations, mutation rates were calculated for spontaneous and BIR mutagenesis using modifications of the Drake equation [69]. Specifically, the rate of spontaneous mutagenesis in experimental strains was calculated using mutation frequencies at 0 h in experimental and no-DSB control strains using the following formula: µ = 0.4343 f/log(Nµ), where µ =  the rate of spontaneous mutagenesis, f =  mutation frequency at time 0 h, and N =  the number of cells in yeast culture at 0 h. Because most strains with a DSB site exhibited residual DSB formation even at 0 h, the rate of spontaneous mutagenesis was more accurately determined from 0 h Lys^+^ frequencies in no-DSB controls using the same formula. For the no-DSB controls with reporters at *MAT*, the median, calculated based on the equation shown above, was divided by 2 to correct for the presence of two *LYS2* reporters in these strains. The rate of mutations after galactose treatment (µ_7_) was determined using a simplified version of the Drake equation: µ_7_ =  (f_7_ − f_0_), where f_7_ and f_0_ are the mutation frequencies at times 7 h and 0 h, respectively. This modification was necessary because experimental strains did not divide between 0 h and 7 h, while no-DSB controls underwent ≤1 division between 0 h and 7 h.

Rates are reported as the median value (Figures 2–4,6), and the 95% confidence limits for the median are calculated for the strains with a minimum of six individual experiments as described and reported in Table S1 [70]. For strains with 4–5 individual experiments, the range of the median was calculated. Statistical comparisons between median mutation rates were performed using the Mann-Whitney U test [71].

### Calculations of BIR Efficiency

BIR efficiency was estimated in all strains with a DSB site, typically in a subset of three experiments per strain. Colonies plated on YEPD 7 h after addition of galactose were replica plated onto omission media to examine the *ADE1*, *ADE3*, and *LEU2* markers. Colonies formed by BIR displayed an Ade^+^Leu^−^, Ade^+/−^Leu^−^, or Ade^+^Leu^+/−^ phenotype [23]. The efficiency of BIR in individual experiments was estimated as the sum of all Ade^+^Leu^−^ events plus one half of all BIR sectored (Ade^+/−^Leu^−^ or Ade^+^Leu^+/−^) events, divided by the total number of colonies analyzed. Typically, ≥50 colonies were analyzed for individual experiments. To compare wild type and mutant strains, BIR efficiency was determined by combining data from isogenic 16 and 36 kb A_4_ strains (strains with the reporter at *MAT* were omitted due to the effect of mating type on BIR efficiency [19]). Medians were compared using the Mann-Whitney U test [71].

### Analysis of Mutation Spectra

A portion of the *LYS2* gene was sequenced from independent Lys^+^ outcomes using one or both of the primers used to confirm insertion of the *LYS2* reporters (see Text S1 for details). For experimental strains undergoing BIR repair, 7 h Lys^+^ BIR events (confirmed as Ade^+^Leu^−^ on selective media) were sequenced. Because these strains did not divide between the 0 h and 7 h time points and the Lys^+^ frequency at 7 h significantly exceeded that at 0 h, all Lys^+^ events resulting from DSB repair were considered independent. In *msh2Δ* A_7_ experimental strains, in which the 0 h rate was extremely elevated, candidates for sequencing were chosen from experiments with a ≥10-fold difference in mutation frequencies between 0 h and 7 h. For no-DSB controls, independent Lys^+^ events were obtained by growing cultures from singles in YEPD overnight and choosing only one event from each culture.

### Determination of dNTP Pools

The methods of measuring dNTPs in yeast are as described in (see Materials and Methods in the Supporting Information section for details) [51]. Results of three time course experiments performed for each strain were used to calculate the average ± standard deviation level of nucleotides.

## Supporting Information

Figure S1 **Spectrum of BIR-associated and spontaneous Lys^+^ mutations in MMR^+^ and** ***msh2Δ*** **strains.** A part of the *LYS2* coding sequence bearing insertion of approximately 61 bp (*Ins*; positions 1–61) is shown. In the sequence, gray box indicates direct repeats flanking the 61 bp insert; ** indicates the location of the A_4_, A_7_, or A_14_ poly-adenine run; underlined, italics indicates the GGGCCAAGG frameshift hotspot (see text for details), for which a partial −1 bp quasipalindromic copy (TTGCCC, also underlined, italics) is located approximately 70 bp away. In the table, numbers indicate 1 bp deletions at the positions depicted on the top; parentheses indicate larger deletions (del) and insertions (ins); “Comp del” indicates reversions to Lys^+^ resulting from complete deletion of *Ins*(A_4_), (A_7_), or (A_14_) that occurred by template switching involving direct repeats (gray boxes) flanking the insertion; “Other” indicates complex events where 1 bp deletions were associated with a nearby base substitution; ^†^ indicates cases where the percentage of −1 bp deletions occurring in the poly-A run is statistically significantly different from the isogenic wild type strain using Fisher's Exact Test (*p*<0.05).
(0.28 MB TIF)

Figure S2 **Effect of UV damage on frameshift mutagenesis.** Lys^+^ frequency was measured in no-DSB controls of wild type and *rev3Δ* mutants containing the A_7_ reporter at the 36 kb position after exposure to 0 or 20 J/m^2^ UV light. * indicates statistically significantly different from no exposure; † indicates statistically different from wild type.
(0.06 MB TIF)

Figure S3 **Growth characteristics of wild type and its** ***pol3-5DVmsh3Δ*** **derivatives.** Strains with the *lys2::Ins(A_4_)* reporter at the 36 kb position were analyzed. (A) FACS analysis of wild type cells before (0 h) and after (1 h, 2 h, 3 h, 4 h, 5 h, 6 h, 7 h) addition of galactose. (B) Growth curve of wild type and its *pol3-5DVmsh3Δ* derivative measured by OD_600_ in YEPD and (C) YEP-Lactate. Each data point represents mean and standard deviation from three independent cultures for each strain.
(0.53 MB TIF)

Figure S4 **BIR-related change in the level of Rnr4p and Rnr2p, and Sml1p.** Levels of Rnr4p, Rnr2p, and Sml1p were analyzed in the wild type and its no-DSB control strains containing the A_4_ reporter at the 16 kb position before (0 h) and after 3 and 6 h of incubation in galactose-containing media. Tubulin levels were used as a loading control. (A) Increased levels of Rnr4p and Rnr2p and decreased levels of Sml1p demonstrate that the DNA damage checkpoint is activated during BIR. Quantification of (B) Rnr4p, (C) Rnr2p, and (D) Sml1p levels using a Fuji LAS-3000 camera and the MultiGauge image analyzing software. Values are normalized to the loading control, tubulin. In (B), the value for No-DSB after 6 h is 0.12, though it is difficult to visualize in the figure.
(0.53 MB TIF)

Table S1 **The rate of spontaneous and DSB-associated Lys^+^ mutations.**
(0.03 MB XLSX)

Table S2 **BIR efficiency in wild type and mutant strains.**
(0.01 MB XLSX)

Text S1 **Supplemental materials and methods.**
(0.06 MB DOC)

## Abbreviations

BIR: break-induced replication
GC: gene conversion
DSB: double-strand break
MMR: mismatch repair
Pol: polymerase
RNR: ribonucleotide reductase
