# Supplementary figures and images for: Break-Induced Replication Is Highly Inaccurate
Source: PLoS Biol. 2011 Feb 15;9(2):e1000594. doi: 10.1371/journal.pbio.1000594 (PMC3039667; doi:10.1371/journal.pbio.1000594)

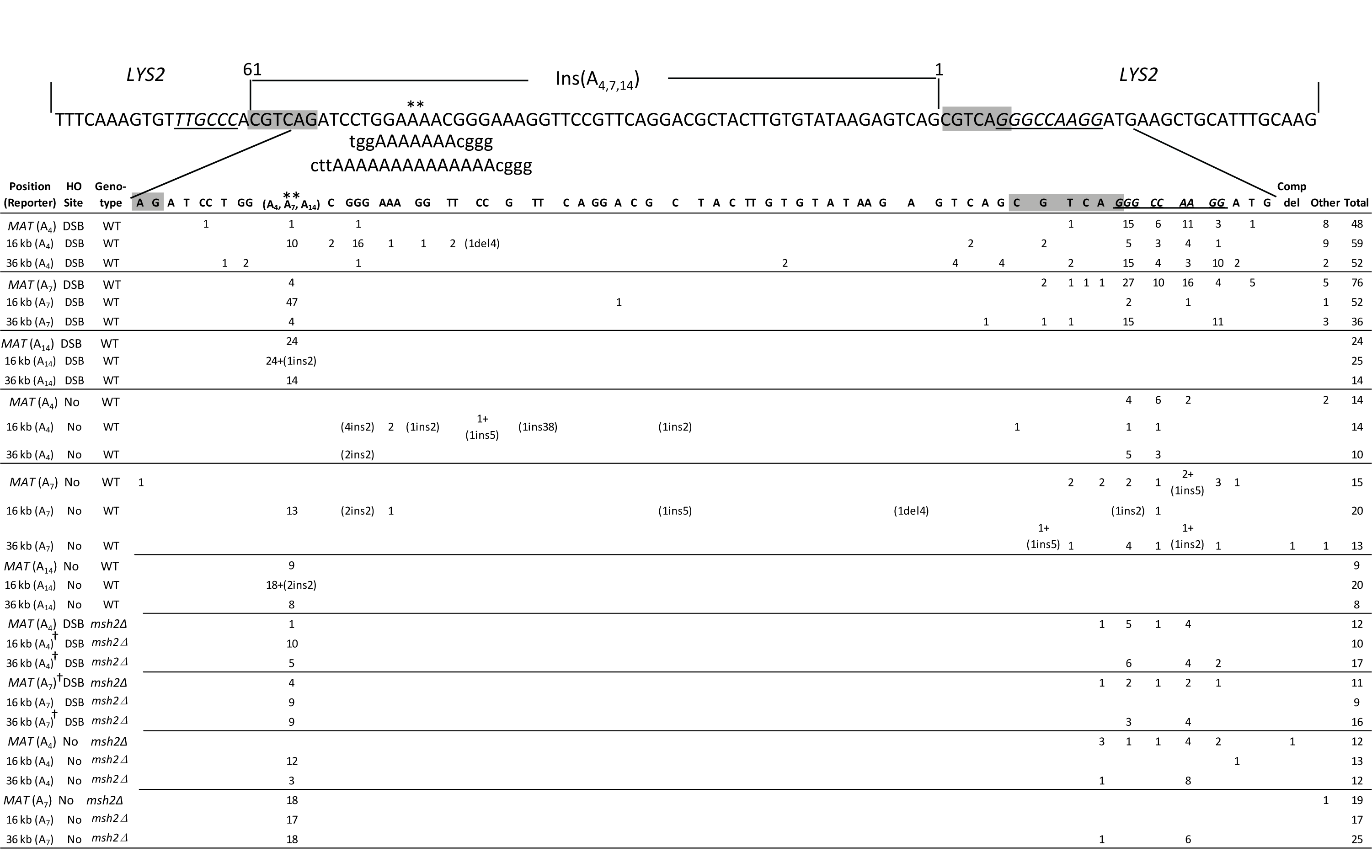

Supplement: Figure S1 — Spectrum of BIR-associated and spontaneous Lys+ mutations in MMR+ and msh2Δ strains. A part of the LYS2 coding sequence bearing insertion of approximately 61 bp (Ins; positions 1–61) is shown. In the sequence, gray box indicates direct repeats flanking the 61 bp insert; ** indicates the location of the A4, A7, or A14 poly-adenine run; underlined, italics indicates the GGGCCAAGG frameshift hotspot (see text for details), for which a partial −1 bp quasipalindromic copy (TTGCCC, also underlined, italics) is located approximately 70 bp away. In the table, numbers indicate 1 bp deletions at the positions depicted on the top; parentheses indicate larger deletions (del) and insertions (ins); “Comp del” indicates reversions to Lys+ resulting from complete deletion of Ins(A4), (A7), or (A14) that occurred by template switching involving direct repeats (gray boxes) flanking the insertion; “Other” indicates complex events where 1 bp deletions were associated with a nearby base substitution; † indicates cases where the percentage of −1 bp deletions occurring in the poly-A run is statistically significantly different from the isogenic wild type strain using Fisher's Exact Test (p<0.05). (0.28 MB TIF) [file pbio.1000594.s001.tif]

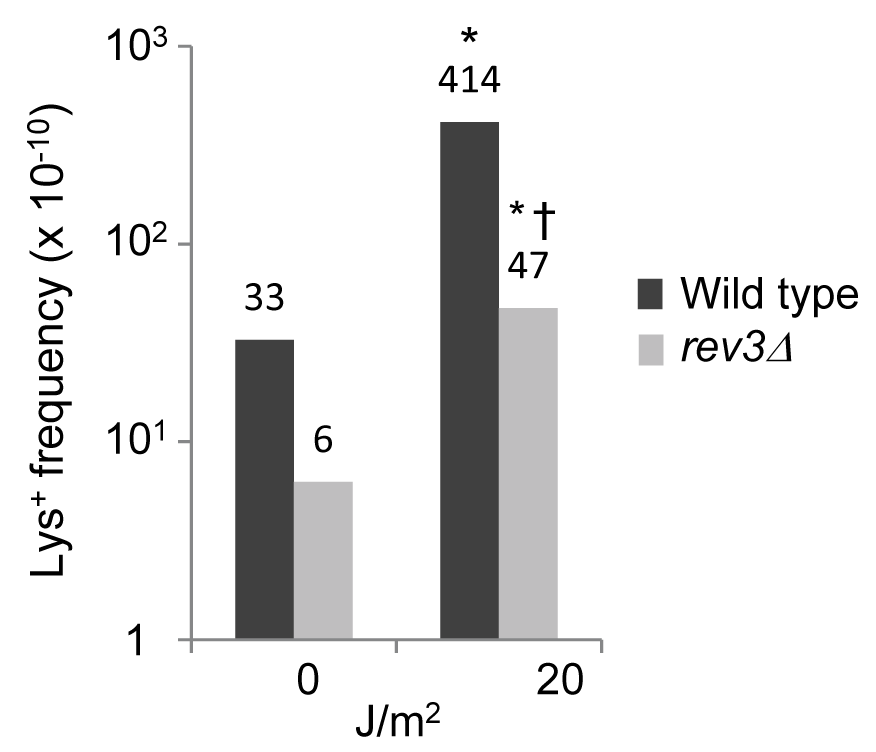

Supplement: Figure S2 — Effect of UV damage on frameshift mutagenesis. Lys+ frequency was measured in no-DSB controls of wild type and rev3Δ mutants containing the A7 reporter at the 36 kb position after exposure to 0 or 20 J/m2 UV light. * indicates statistically significantly different from no exposure; † indicates statistically different from wild type. (0.06 MB TIF) [file pbio.1000594.s002.tif]

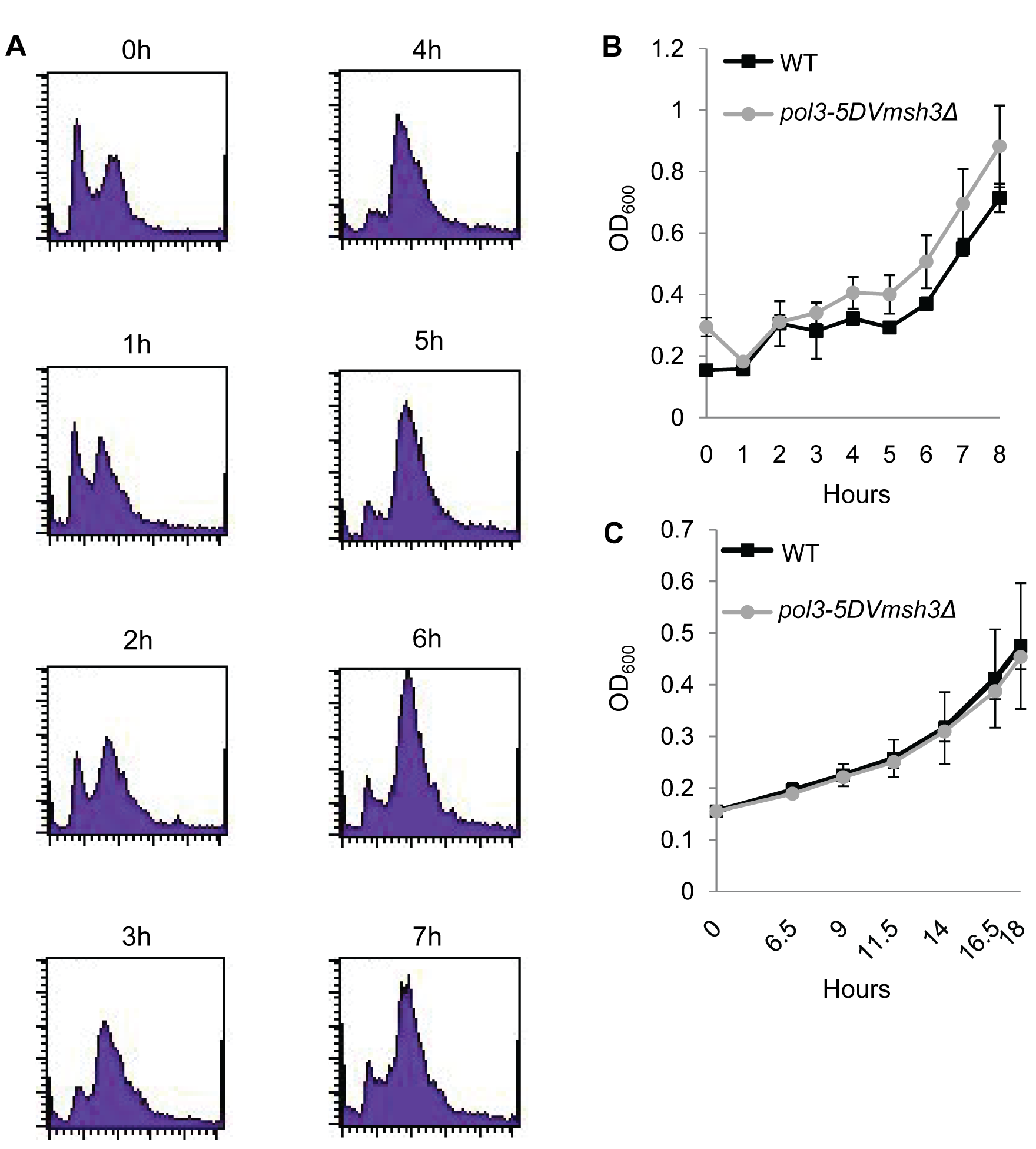

Supplement: Figure S3 — Growth characteristics of wild type and its pol3-5DVmsh3Δ derivatives. Strains with the lys2::Ins(A4) reporter at the 36 kb position were analyzed. (A) FACS analysis of wild type cells before (0 h) and after (1 h, 2 h, 3 h, 4 h, 5 h, 6 h, 7 h) addition of galactose. (B) Growth curve of wild type and its pol3-5DVmsh3Δ derivative measured by OD600 in YEPD and (C) YEP-Lactate. Each data point represents mean and standard deviation from three independent cultures for each strain. (0.53 MB TIF) [file pbio.1000594.s003.tif]

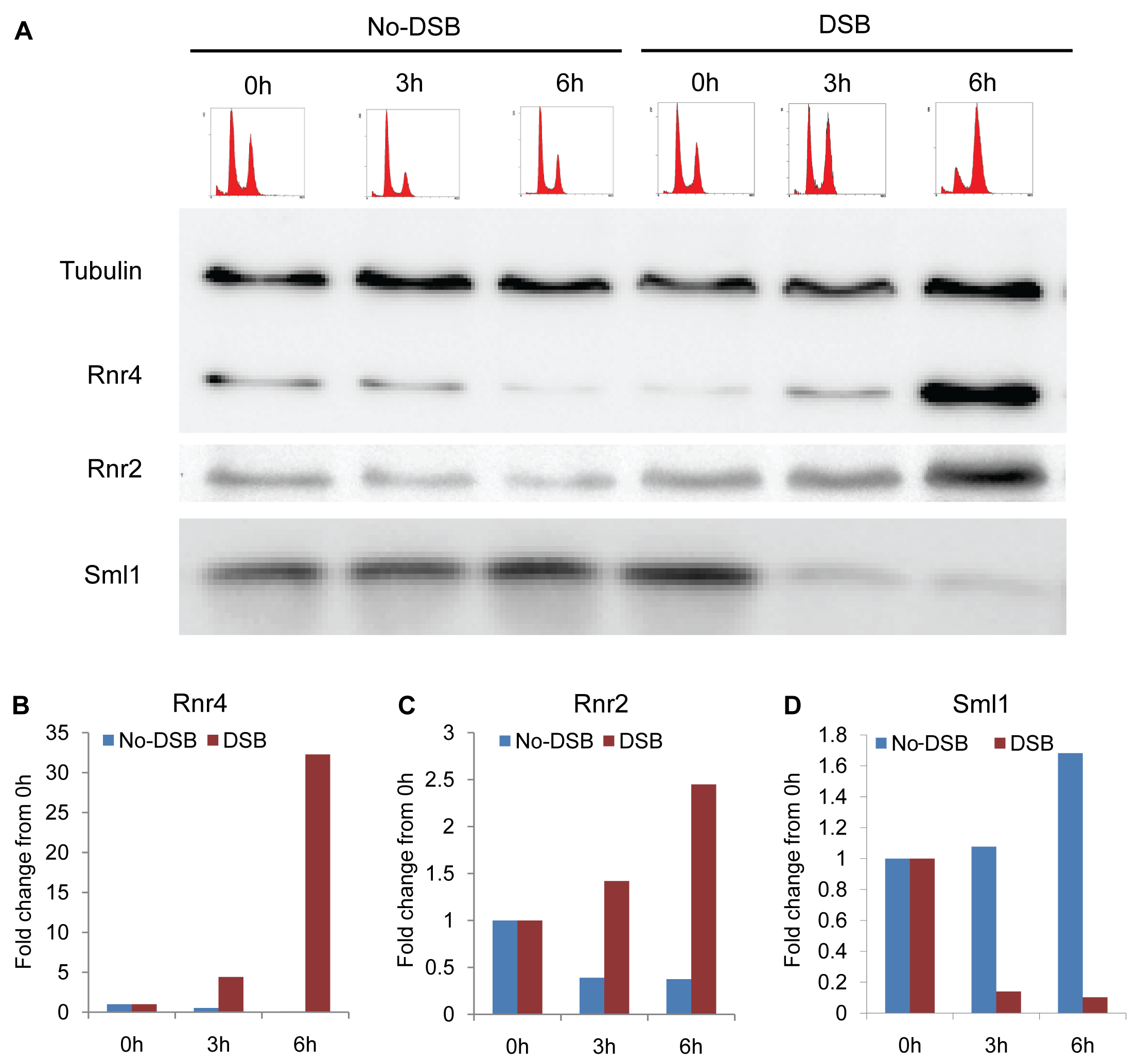

Supplement: Figure S4 — BIR-related change in the level of Rnr4p and Rnr2p, and Sml1p. Levels of Rnr4p, Rnr2p, and Sml1p were analyzed in the wild type and its no-DSB control strains containing the A4 reporter at the 16 kb position before (0 h) and after 3 and 6 h of incubation in galactose-containing media. Tubulin levels were used as a loading control. (A) Increased levels of Rnr4p and Rnr2p and decreased levels of Sml1p demonstrate that the DNA damage checkpoint is activated during BIR. Quantification of (B) Rnr4p, (C) Rnr2p, and (D) Sml1p levels using a Fuji LAS-3000 camera and the MultiGauge image analyzing software. Values are normalized to the loading control, tubulin. In (B), the value for No-DSB after 6 h is 0.12, though it is difficult to visualize in the figure. (0.53 MB TIF) [file pbio.1000594.s004.tif]
