# Supplementary material for: Break-Induced Replication Is Highly Inaccurate
Source: PLoS Biol. 2011 Feb 15;9(2):e1000594. doi: 10.1371/journal.pbio.1000594 (PMC3039667; doi:10.1371/journal.pbio.1000594)
Supplement: Text S1 — Supplemental materials and methods. (0.06 MB DOC) [file pbio.1000594.s007.doc]

**Supplemental materials and methods**

Yeast strains to measure BIR-associated mutagenesis.

First, AM1229 was constructed by deleting the *LYS2* gene in AM1003 from its native position on chromosome II by the *delitto perfetto* protocol [[1](#_ENREF_1),[2](#_ENREF_2)], which involved two steps. Initially, AM1257 was constructed by transformation of AM1003 with a DNA fragment generated by PCR amplification of pCORE using the following primers in which uppercase letters correspond to targeting tails and lowercase letters are pCORE-amplification sequences: 5’‑CCACGAGCACCAGCACCTGAAGCAACTAGACTTATTTGCGCTTGAGTTAGgagctcgttttcgacactgg‑3’ and 5’‑GGTCTGGATAGAGAAGTTGGATAATCCAACTCTTTCAGTGTTACCACATGtccttaccattaagttgatc‑3’. Subsequently, AM1229 was constructed by transformation of AM1257 with a mixture of two oligonucleotides containing complementary sequences that corresponded to positions upstream and downstream of *LYS2*. The oligonucleotides used, with complementarity indicated in uppercase or lowercase letters, were: 5’‑CATATTTAATTATTGTACATGGACATATCATACGTAATGCTCAACCTgcaagtggaattccgctggcaaactattgaagagttttcctcgc‑3’ and 5’‑gcgaggaaaactcttcaatagttttgccagcggaattccacttgcAGGTTGAGCATTACGTATGATATGTCCATGTACAATAATTAAATATG‑3’. Second, AM1248 was constructed by transformation of AM1229 with a DNA fragment generated by PCR amplification of *THR4* to create a Thr^+^ strain. The primers used for the amplification were: 5’‑TGCTACCACCTTGGATGTAGATGT‑3’ and 5’‑GTGTCATCGATGATAATGATCCGT‑3’.

Third, three derivatives of AM1248 with the *LYS2* ORF inserted at three different positions of the *MATα-inc* containing chromosome were created. The primers with targeting tails (uppercase) and *LYS2*-amplification sequence (lowercase) to create the three strains were: 1) To create AM1355 with *LYS2* in the *MATα-inc* gene (“*MAT*” position), 5’‑TTTATCATATCTTGAGTTACCACATTAAATACCAACCCATCCGCCGATTTaattacataaaaaattccggcgg‑3’ and 5’‑TTCAGCGAGCAGAGAAGACAAGACATTTTGTTTTACACCGGAGCCAAACTGaagctgctgcggagcttcc‑3’; 2) To create AM1247 with *LYS2* at the 16-kb position inserted between *RSC6* and *THR4*, 5’‑GAGTAGTGACCGTGCGAACAAAAGAGTCATTACAACGAGGAAATAGAAGAaattacataaaaaattccggcgg‑3’ and 5’‑ATATAAGATACACAATATAGATAGTATTAAAAAAACGTGTATACGTTATTttaagctgctgcggagcttcc‑3’; and 3) To create AM1284 with *LYS2* at the 36-kb position between *SED4* and *ATG15*, 5’‑AAATCGTAAATACATAGGCTGGGCCATATACACTAACATGTGTCGTGACCAttaagctgctgcggagcttcc‑3’ and 5’‑TTATTTTCTTTCCGATGTTATGCTTATTATATCTGTGATTGATAAGAGAAaattacataaaaaattccggcgg‑3’. Finally, the *lys2(A_4_)*, *lys2(A_7_)*, or *lys2(A_14_)* frameshift reporters were inserted into each of these constructs using methods described in [[3](#_ENREF_3)] and confirmed by sequencing with the following primers: 5’‑GTTCGTACCCCTCTCGAGAATA‑3’ and 5’‑ATTTGAGGCAAATTTTTCGTTCCAA‑3’.

Determination of dNTP pools

Cells were grown in YEP media (2% yeast extract, 1% Bacto peptone) supplemented with 2% raffinose to logarithmic phase, then *Gal::HO* was induced by adding 20% galactose to a final concentration of 2%. 3.70x10^8^ (as determined by OD_600_) cells were harvested before induction and after 3h and 6h by filtration through 25mm White AAWP nitrocellulose filters (0.8 μm, Millipore AB, Solna, Sweden). The filters were immersed in 700 μL of ice-cold extraction solution (12% w/v trichloroacetic acid, 15mM MgCl_2_) in Eppendorf tubes. The following steps were carried out at 4° C. The tubes were vortexed for 30 s, incubated for 15 min and vortexed again for 30 s. The filters were removed and the supernatants were collected after centrifugation at 20,000g for 1 min and added to 800 μL of ice-cold Freon–trioctylamine mixture [10 mL of Freon (1,1,2-trichlorotrifluoroethane), Aldrich, Sigma-Aldrich Sweden AB, Stockholm, Sweden, 99% and 2.8 mL of trioctylamine, Fluka, Sigma-Aldrich Sweden AB, Stockholm, Sweden, >99%]. The samples were vortexed and centrifuged for 1 min at 20,000g. The aqueous phase was collected and added to 700 μL of ice-cold Freon–trioctylamine mixture. 475 and 47.5 μL of the aqueous phase were collected. The 475 μL aliquots of the aqueous phase were pH adjusted with 1M NH_4_HCO_3_ (pH 8.9), loaded on boronate columns [Affi-Gel 601 (Bio-Rad)] and eluted with 50mM NH_4_HCO_3_, pH 8.9, 15mM MgCl_2_ to separate dNTPs and NTPs. The eluates with purified dNTPs were adjusted to pH 3.4 with 6M HCl, separated on a Partisphere SAX-5 HPLC column (4.6 mm x125 mm, Whatman International Ltd.) and quantified using a UV-2075 Plus detector (Jasco, Mölndal, Sweden). Nucleotides were isocratically eluted using 0.36M ammonium phosphate buffer (pH 3.4, 2.5% v/v acetonitrile). The 47.5 μL aliquots of the aqueous phase were adjusted to pH 3.4 and used to quantify NTPs by HPLC in the same way as dNTPs. Results from dNTP measurements were normalized to NTP levels of the cells.

Western blotting

Protein samples for Western blotting were prepared as described [[4](#_ENREF_3)]. Proteins were separated by SDS-PAGE and transferred to a nitrocellulose membrane (Protran BA 85, Whatman) using the Minigel System (C.B.S. Scientific Co.). Detection of Rnr2p and Sml1p was done using affinity-purified rabbit polyclonal antibodies produced by Agrisera, Sweden. For simultaneous detection of both Rnr4 and α-tubulin , the YL1/2 rat monoclonal antibodies (Santa Cruz Biotechnology) were used at 1:500.

Flow cytometry

Samples for flow cytometry presented in Fig. S3A were prepared as described in and analyzed on a Becton Dickinson FACScan flow cytometer. Flow cytometry presented in Fig. S4A was performed as described in .

Growth rates

Growth of strains in YEPD was measured by OD_600_ (SpectraMax M2, Molecular Devices) after 2 mL of saturated inoculum was diluted into 50 mL of YEPD medium. Cells were allowed to adjust to new media for 2 hours prior to measuring the 0h timepoint. Growth of strains in YEP-Lac was measured as described for YEPD immediately after 2 mL of saturated inoculum was diluted into 50 mL of YEP-Lac medium and followed for 18 hours.

**Supplemental references**

1. Storici F, Lewis LK, Resnick MA (2001) *In vivo* site-directed mutagenesis using oligonucleotides. Nat Biotechnol 19: 773-776.

2. Storici F, Resnick MA (2006) The *delitto perfetto* approach to *in vivo* site-directed mutagenesis and chromosome rearrangements with synthetic oligonucleotides in yeast. Methods Enzymol 409: 329-345.

3. Tran HT, Keen JD, Kricker M, Resnick MA, Gordenin DA (1997) Hypermutability of homonucleotide runs in mismatch repair and DNA polymerase proofreading yeast mutants. Mol Cell Biol 17: 2859-2865.

4. Standart NM, Bray SJ, George EL, Hunt T, Ruderman JV (1985) The small subunit of ribonucleotide reductase is encoded by one of the most abundant translationally regulated maternal RNAs in clam and sea urchin eggs. The Journal of Cell Biology 100: 1968-1976.

5. Malkova A, Naylor ML, Yamaguchi M, Ira G, Haber JE (2005) *RAD51*-dependent break-induced replication differs in kinetics and checkpoint responses from *RAD51*-mediated gene conversion. Mol Cell Biol 25: 933-944.

6. Chabes A, Stillman B (2007) Constitutively high dNTP concentration inhibits cell cycle progression and the DNA damage checkpoint in yeast *Saccharomyces cerevisiae*. Proc Natl Acad Sci U S A 104: 1183-1188.
